# The *PALB2* p.Leu939Trp mutation is not associated with breast cancer risk

**DOI:** 10.1186/s13058-016-0762-9

**Published:** 2016-11-09

**Authors:** Irene Catucci, Paolo Radice, Roger L. Milne, Fergus J. Couch, Melissa C. Southey, Paolo Peterlongo

**Affiliations:** 1 IFOM, Fondazione Istituto FIRC di Oncologia Molecolare, via Adamello 16, 20139 Milan, Italy; 2 Unit of Molecular Bases of Genetic Risk and Genetic Testing, Department of Preventive and Predictive Medicine, Fondazione IRCCS Istituto Nazionale dei Tumori, Milan, Italy; 3 Cancer Epidemiology Centre, Cancer Council Victoria, Melbourne, Victoria Australia; 4 Centre for Epidemiology and Biostatistics, Melbourne School of Population and Global Health, University of Melbourne, Victoria, Australia; 5 Department of Laboratory Medicine and Pathology, Mayo Clinic, Rochester, Minnesota USA; 6 Genetic Epidemiology Laboratory, Department of Pathology, The University of Melbourne, Parkville, Australia

**Keywords:** Breast cancer predisposition, Breast cancer genetic risk factor, *PALB2* p.Leu939Trp, VUS

## Text

Missense mutations in breast cancer predisposition genes are a substantial clinical problem. These are usually considered variants of uncertain significance (VUS) until genetic, clinical, and functional data provide statistical evidence for reclassification as pathogenic or neutral.

Recently, Park et al. [1] suggested that the WD40 domain of the protein encoded by the breast cancer predisposition gene *PALB2* may scaffold RAD51C, RAD51, and BRCA2 proteins into a complex involved in DNA repair mediated by homologous recombination (HR). The authors studied the effect of p.Leu939Trp and other missense mutations located within the *PALB2* WD40 domain that had been identified in the germline of women with breast cancer. They reported that the p.Leu939Trp mutation resulted in altered PALB2–BRCA2 binding, decreased capacity for DNA double-strand break-induced HR, and increased sensitivity to ionizing radiation. Based on these observations and their assertion that this mutation occurs more frequently in women with breast cancer than in unaffected women, Park et al. [1] concluded that the p.Leu939Trp mutation may be pathogenic and proposed that their assays could be used for the functional characterization of other *PALB2* missense variants.

**Fig. 1:**
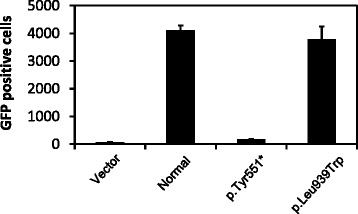
Homologous recombination assay for the p.Leu939Trp mutation. B400 palb2−/−; tp53−/− mouse mammary tumor cells were co-transfected with direct repeat-green fluorescent protein (DR-GFP) reporter and recombinant constructs expressing normal (used as positive control), p.Tyr551* (used as negative control) and p.Leu939Trp mutated PALB2 alleles. The error bars represent the Standard Error (SE) of the mean from three independent experiments. GFP-positive cells were assessed by flow cytometry. Comparable expression level of normal and p.Leu939Trp mutated PALB2 proteins was observed by western blot (data not shown)

Case-control, rather than case-only studies are required to estimate the relative risk associated with a genetic variant. Park et al. refer to p.Leu939Trp as a breast cancer-associated mutation; however, among the studies they cited to sustain this hypothesis, only one was a case-control study [2]. Further, case-control data from Rahman et al. [3] were not considered, even though this study is referenced in their report. These two studies [2, 3] together identified the p.Leu939Trp mutation in 10/1741 (0.57 %) women with breast cancer and 8/1534 (0.52 %) unaffected controls, suggesting that this mutation is not associated with breast cancer risk. Further, we later published a third study corroborating this null finding [4] and have subsequently reported additional evidence that the p.Leu939Trp mutation is not associated with breast cancer risk, based on genotyping of 42,671 breast cancer cases and 42,164 controls (odds ratio = 1.05, 95 % confidence interval = 0.83–1.32, *p* value = 0.70) [5]. Finally, we observed that the p.Leu939Trp mutation does not disrupt the HR-mediated DNA repair activity of PALB2 (Fig. 1).

Results from functional assays with undefined sensitivity and specificity are not sufficient to classify VUS. In this instance, the p.Leu939Trp mutation may have some influence on response to ionizing radiation but it appears to have little to no impact on HR-mediated DNA repair. In conclusion, our findings suggest that the *PALB2* p.Leu939Trp mutation should be classified as a neutral variant with no clinical relevance to risk of breast cancer.
